# Transcriptional response of *Hoxb* genes to retinoid signalling is regionally restricted along the neural tube rostrocaudal axis

**DOI:** 10.1098/rsos.160913

**Published:** 2017-04-05

**Authors:** Nicoletta Carucci, Emanuele Cacci, Paola S. Nisi, Valerio Licursi, Yu-Lee Paul, Stefano Biagioni, Rodolfo Negri, Peter J. Rugg-Gunn, Giuseppe Lupo

**Affiliations:** 1 Department of Biology and Biotechnology ‘C. Darwin’, Sapienza University of Rome, 00185 Rome, Italy; 2 Istituto Pasteur— Fondazione Cenci Bolognetti, Sapienza University of Rome, 00185 Rome, Italy; 3 Department of Chemistry, Sapienza University of Rome, 00185 Rome, Italy; 4 Epigenetics Programme, The Babraham Institute, Cambridge CB22 3AT, UK

**Keywords:** neural stem/progenitor cells, retinoic acid, *Hoxb* genes, epigenetic regulation, telencephalon, spinal cord

## Abstract

During vertebrate neural development, positional information is largely specified by extracellular morphogens. Their distribution, however, is very dynamic due to the multiple roles played by the same signals in the developing and adult neural tissue. This suggests that neural progenitors are able to modify their competence to respond to morphogen signalling and autonomously maintain positional identities after their initial specification. In this work, we take advantage of *in vitro* culture systems of mouse neural stem/progenitor cells (NSPCs) to show that NSPCs isolated from rostral or caudal regions of the mouse neural tube are differentially responsive to retinoic acid (RA), a pivotal morphogen for the specification of posterior neural fates. *Hoxb* genes are among the best known RA direct targets in the neural tissue, yet we found that RA could promote their transcription only in caudal but not in rostral NSPCs. Correlating with these effects, key RA-responsive regulatory regions in the *Hoxb* cluster displayed opposite enrichment of activating or repressing histone marks in rostral and caudal NSPCs. Finally, RA was able to strengthen *Hoxb* chromatin activation in caudal NSPCs, but was ineffective on the repressed *Hoxb* chromatin of rostral NSPCs. These results suggest that the response of NSPCs to morphogen signalling across the rostrocaudal axis of the neural tube may be gated by the epigenetic configuration of target patterning genes, allowing long-term maintenance of intrinsic positional values in spite of continuously changing extrinsic signals.

## 1. Introduction

A remarkable feature of vertebrate neural development is the early subdivision of the neuroectoderm along its rostrocaudal and dorsoventral axes into an orderly array of sharply delimited domains expressing region-specific combinations of transcription factor-encoding genes [1,2]. For example, expression of *Otx1/2* genes identifies presumptive anterior (forebrain and midbrain) territories [3], whereas *Hox* genes are expressed within posterior (hindbrain and spinal cord) neuroectoderm [4,5]. Other gene families define positional identities along the neural tube dorsoventral axis [2]. Eventually, various combinations of transcription factors become expressed in different areas of the neural tube, leading to specification of cellular compartments with distinct transcriptional programmes and developmental fates [6].

Transcription factors involved in the specification of positional identities acquire their spatially limited expression domains in response to the action of a number of diffusible morphogens that are distributed with concentration gradients across the neural tissue [2,7,8]. Among them, retinoic acid (RA) is a key morphogen controlling rostrocaudal neural patterning [7,9,10]. RA is a product of vitamin A metabolism that is present with a posterior-high to anterior-low gradient in early vertebrate embryos due to the presence of RA-synthesizing enzymes (such as Aldh1a2) in caudal regions and RA-degrading enzymes (such as Cyp26a1) in anterior regions [9–11]. The posterior neural tube develops abnormally in embryos with reduced levels of RA [12–15]. Furthermore, embryos exposed to exogenous RA during early developmental stages undergo dramatic losses of forebrain structures and expansion of posterior neural structures [16–18]. RA regulates gene transcription by binding retinoic acid receptors (RARs), which interact with retinoic acid response elements (RAREs) within regulatory regions of RA-responsive genes to control their expression [11]. In particular, well-characterized RAREs are present within *Hox* gene clusters [19,20] and transcription of *Hox* genes is readily upregulated following RA treatments of early vertebrate embryos or *in vitro* cellular models of early embryonic cells [17,21–25]. Hence, RA can promote posterior neural fates by directly activating *Hox* gene expression [26], while repressing, directly or indirectly, transcription of *Otx2* and other rostral specification genes [17,27].

Although the presumptive anterior neuroectoderm is very sensitive to the caudalizing activity of RA during gastrulation, vertebrate embryos exposed to exogenous RA at later developmental stages become progressively resistant to suppression of anterior development [17,18,28]. Furthermore, at these later stages, endogenous retinoid signalling is essential for proper forebrain formation. For example, RAR-dependent signalling has been implicated in patterning and morphogenesis of the optic cup [29–32], in the differentiation of GABAergic neurons in the basal ganglia of the telencephalon [33], and in the control of neuronal differentiation and migration in the developing cerebral cortex [34–37]. Retinoid signalling also appears to control neural progenitor differentiation in the subventricular zone (SVZ) and dentate gyrus neurogenic niches of the postnatal mammalian forebrain [38,39]. These observations suggest that anterior neural progenitors drastically modify their responsiveness to retinoid signalling during development, losing competence in retinoid-dependent caudalization while becoming able to provide alternative, cell type-specific responses following exposure to locally produced retinoids. Nonetheless, direct evidence that neural progenitors in the embryonic and postnatal forebrain are effectively refractory to posteriorization by retinoid signalling is still lacking. Moreover, it remains unclear how the intrinsic competence of forebrain progenitors to acquire posterior positional identities may be restricted at the molecular level.

Previous studies have shown that neural stem/progenitor cells (NSPCs) derived from the embryonic and the adult murine central nervous system (CNS) can be extensively propagated *in vitro* using appropriate adherent culture conditions that allow long-term self-renewal [40,41]. Furthermore, NSPCs obtained from different rostrocaudal regions of the mouse neural tube at embryonic day 13.5–14.5 (E13.5–14.5) or from the adult mouse forebrain maintain distinct expression profiles of transcription factor-encoding genes that are consistent with the respective areas of derivation [42,43]. These transcriptional profiles persist even after several passages in culture, suggesting that NSPCs can retain, at least in part, their positional identities *in vitro* [42,43]. In this work, we took advantage of this experimental system to demonstrate that NSPCs derived from both rostral and caudal regions of E13.5 mouse neural tube can activate retinoid signalling when exposed to RA, but they respond differently to it. In particular, exogenous RA can promote expression of genes of the *Hoxb* cluster in posterior, but not in anterior NSPCs, which remain resistant to RA-dependent *Hoxb* transcription also during adulthood and ageing. We further show that *Hoxb* chromatin, including key RAREs, is differentially enriched for activating and repressing histone modifications in rostral and caudal NSPCs. In agreement with transcriptional effects, RA treatments are unable to reverse *Hoxb* chromatin repression in rostral NSPCs, but can enhance *Hoxb* chromatin activation in caudal NSPCs.

Altogether, these results suggest that intrinsic epigenetic differences at the level of key regionally expressed genes may constrain transcriptional responses to morphogen signalling and safeguard maintenance of rostrocaudal positional identities in NSPCs.

## 2. Material and methods

### 2.1. Mouse neural stem/progenitor cell culture

NSPCs derived from mouse E13.5 cerebral cortex or spinal cord and protocols for their *in vitro* culture were previously described [43]. Cortex NSPCs were obtained from the dorsolateral wall of the telencephalon, corresponding to the developing cerebral cortex. Spinal cord NSPCs were derived from spinal cord tissue dissected between the hindbrain/spinal cord boundary and hindlimb buds. Adult or aged SVZ NSPCs were derived from 3-month- or 18-month-old mice, respectively, and cultured *in vitro* as previously described [44]. NSPCs were routinely expanded in T25 flasks (Corning) that were coated with 10 µg ml^−1^ poly-ornithine (Sigma-Aldrich) and 5 µg ml^−1^ laminin (Sigma-Aldrich), using previously described chemically defined media [43,44] supplemented with 20 ng ml^−1^ human recombinant epidermal growth factor (R&D Systems) and 10 ng ml^−1^ human recombinant fibroblast growth factor-basic (Peprotech). Media for embryonic cortex and spinal cord NSPC culture also contained 1 : 100 N2 supplement (Invitrogen), while 1 : 50 B27 supplement minus vitamin A (Invitrogen) was used for adult SVZ NSPC cultures. NSPCs were passaged every 3–5 days using Accutase (Sigma-Aldrich) and usually seeded at a density of 10–20 000 cells cm^−2^. NSPCs expanded for up to 20 passages *in vitro* since their initial derivations were used for this work.

### 2.2. Luciferase assays

To detect activation of retinoid signalling in NSPC cultures treated with RA, 2 × 10^6^ embryonic cortex or spinal cord NSPCs were transfected with 2 µg of tk-(βRARE)_2_-luc plasmid, in which expression of firefly luciferase is under control of RAREs [12], and 100 ng of pRL-TK plasmid, which drives constitutive expression of *Renilla* luciferase as a control for transfection efficiency [45]. NSPC transfection was carried out using an Amaxa mouse neural stem cell Nucleofector kit (Lonza) on an Amaxa Nucleofector device (Lonza). Transfected cells were seeded in one six-well plate (Corning). The day after transfection, half of the wells received fresh media supplemented with all-trans RA (Sigma-Aldrich) diluted from a 25 mM stock in DMSO. Media added to the other half contained equal volumes of DMSO. After 24 h of treatment, cells were harvested and reporter expression levels were measured using the dual-luciferase reporter assay system (Promega) on a GloMax multi+ detection device (Promega) as previously described [45].

### 2.3. Real-time RT-PCR

For gene expression analysis by real-time RT-PCR in RA-treated cultures, embryonic cortex or spinal cord NSPCs were seeded in six-well plates (Corning) at a density of 10 000 cells cm^−2^. Adult SVZ NSPCs were instead seeded in T25 flasks at the same density. After 24 h, cultures received fresh media containing appropriate concentrations of RA or DMSO solvent. Media supplemented with RA or DMSO were again replaced the next day and cultures were harvested for molecular analysis after 48 h of treatment.

Total RNA was extracted from frozen cell pellets using the Qiagen RNeasy mini kit and quantified with a NanoDrop 2000 (Thermo Scientific). For real-time RT-PCR, RNA was reverse-transcribed using the Qiagen QuantiTect reverse transcription kit and amplified on a Rotor-Gene Q (Qiagen), using the Qiagen QuantiFast SYBR Green PCR kit. Primers for real-time RT-PCR were either purchased from Qiagen or designed using Primer3 (http://bioinfo.ut.ee/primer3/). Primer sequences selected with Primer3 are listed in the electronic supplementary material, table S1. Relative gene expression levels in different samples were determined with the built-in comparative quantitation method [46], using *Eef1a1* or *Rpl19* as reference genes, with similar results. Statistical analysis of experimental data was carried out using the Microsoft Excel software.

### 2.4. Chromatin immunoprecipitation

For detection of histone H3 modifications by chromatin immunoprecipitation (ChIP), NSPCs were seeded in 100 mm plates at a density of 12–18 000 cells cm^−2^, until cultures reached approximately 80% confluence. Usually, 10–12 × 10^6^ cells for each experimental sample were used in the following steps. At the time of harvesting, cultures were rinsed with PBS, followed by cross-linking with 1% formaldehyde in PBS for 10 min and quenching with 125 mM glycine for 5 min. Cells were then washed with PBS, harvested with a cell scraper in PBS, centrifuged and lysed in lysis buffer (5 mM PIPES, 85 mM KCl, 0.5% NP-40) for 20 min on ice. This was followed by centrifugation, pellet resuspension in shearing buffer (50 mM Tris (pH 8.1), 10 mM EDTA, 0.1% SDS, 0.5% sodium deoxycholate), sonication to an approximate size of 250–500 bps and pre-clearing using Dynabeads-protein G (Invitrogen). The protein content of pre-cleared chromatin was measured using the Bradford assay and 25 µg aliquots of each sample were used for immunoprecipitation with the following antibodies (Cell Signaling): anti-trimethyl-lysine 27 of histone H3 (C36B11), anti-trimethyl-lysine 4 of histone H3 (C42D8), anti-acetyl-lysine 9/14 of histone H3. Samples were incubated overnight at 4°C with approximately 1–5 µg ml^−1^ of each antibody in modified RIPA buffer (140 mM NaCl, 10 mM Tris (pH 7.5), 1 mM EDTA, 0.5 mM EGTA, 1% Triton X-100, 0.01% SDS, 0.1% sodium deoxycholate). As a negative control, an equivalent concentration of normal rabbit IgG (Cell Signaling) was used. Immunocomplexes were recovered by incubating each sample with 20 µl of Dynabeads-protein G for 90 min at 4°C, followed by a series of washes and elution with 0.1 M NaHCO_3_, 1% SDS buffer. All steps between cross-linking and elution included protease inhibitor cocktail (Sigma-Aldrich). Eluted chromatin was treated with RNase A and proteinase K, and immunoprecipitated DNA was purified using the Nucleospin gel and PCR clean-up kit (Macherey Nagel), along with DNA from 20 µl of pre-cleared, non-immunoprecipitated chromatin for each sample (input sample). Purified DNA from immunoprecipitated and input samples was used for real-time PCR analysis as described above. Primers for real-time ChIP-PCR were designed using Primer3 and are listed in the electronic supplementary material, table S2. Amplification levels in immunoprecipated samples relative to the corresponding input samples were measured and normalized to a reference amplicon located approximately 1 kb 5' of *Eef1a1*.

## 3. Results

### 3.1. Retinoid signalling can upregulate *Hoxb* gene expression in spinal cord, but not in telencephalic NSPCs

Previous work showed that NSPCs derived from different districts of mouse E13.5–14.5 neural tube retain position-specific gene expression profiles during *in vitro* culture that are consistent with the region of derivation [42,43]. In this study, we investigated whether retinoid signalling, a key pathway in the specification of posterior neural fates [7,9,10], could challenge the positional identity of NSPCs derived from the rostral (telencephalon) neural tube region, in comparison with caudal (spinal cord-derived) NSPCs.

**Figure 1.**
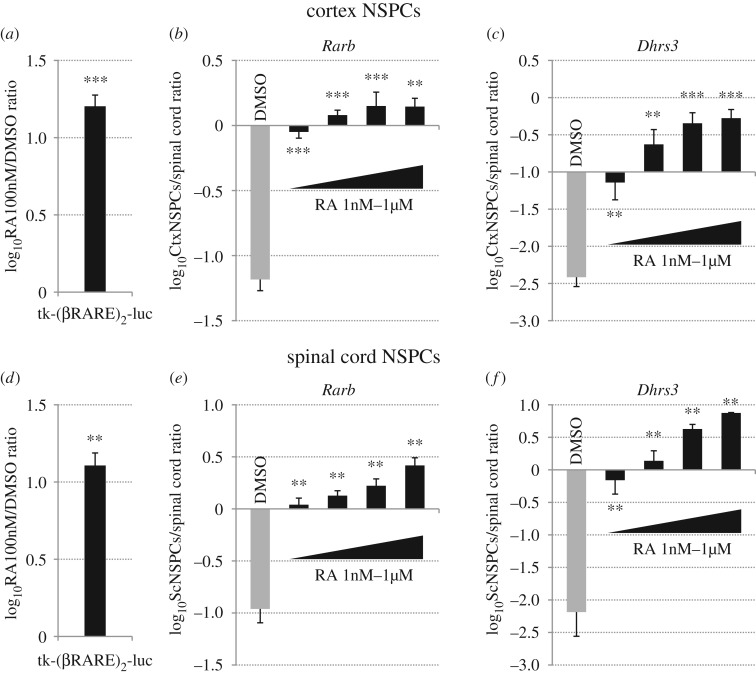
Retinoid signalling is functional in cortex and spinal cord NSPCs and it can be efficiently activated by exogenous RA. (*a*,*d*) Luciferase reporter assays in cortex (*a*) or spinal cord (*d*) NSPCs that were electroporated with an RA-responsive luciferase-expressing plasmid (tk-(βRARE)_2_-luc) and treated with DMSO or 100 nM RA for 24 h. Reporter activity is significantly increased in both cell types at comparable levels by RA treatments. Results are shown as the mean of the log_10_-transformed ratio between RA and DMSO conditions in three to four biological replicates. (*b*,*c*,*e*,*f*) Real-time RT-PCR quantification of gene expression in cortex NSPCs (CtxNSPCs) (*b*,*c*) or spinal cord NSPCs (ScNSPCs) (*e*,*f*) that were treated with either DMSO or 1, 10, 100 or 1000 nM RA for 48 h, showing that RA treatments efficiently upregulate the RA-responsive genes *Rarb* and *Dhrs3* in both cell types. Results are shown as the mean of the log_10_-transformed ratio between DMSO-treated or RA-treated NSPCs and E13.5 spinal cord tissue in four to five biological replicates. In (*a*–*f*), error bars show s.e.m. **p < 0.01; ***p < 0.001 according to a two-tailed Student's *t*-test performed between DMSO and RA conditions.

To this aim, we first verified whether retinoid signalling is functional and can be activated by exposure to exogenous RA both in rostral and in caudal NSPCs. Previously described murine NSPC cultures derived from E13.5 cerebral cortex or spinal cord [43] were transfected with RA-responsive tk-(βRARE)_2_-luc luciferase reporter plasmid [12], as described in the Material and methods section. The day after transfection, NSPCs were treated with DMSO or 100 nM RA and harvested 24 h later for luciferase detection. As shown in figure 1*a*,*d*, RA treatments upregulated reporter expression at similar levels in cortex and spinal cord NSPCs. In addition, we analysed RA effects on transcription of *Rarb*, *Dhrs3* and *Cyp26a1*, which are well-described retinoid-responsive genes in various tissues [47–50], by real-time RT-PCR. Treatments with different concentrations of RA ranging from 1 nM to 1 µM for 48 h caused robust, dose-dependent upregulation of all these genes both in cortex and in spinal cord NSPCs (figure 1*b*,*c*,*e*,*f*; electronic supplementary material, figure S1*a* and S1*d*).

**Figure 2.**
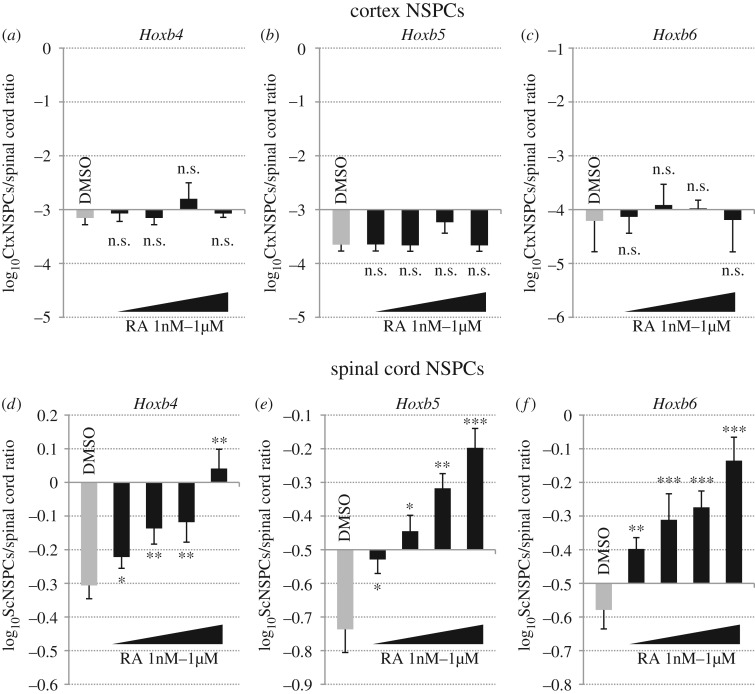
*Hoxb4–6* genes are differentially expressed in cortex and spinal cord NSPCs and are responsive to retinoid signalling only in spinal cord NSPCs. (*a*–*f*) Real-time RT-PCR quantification of *Hoxb4*, (*a*,*d*), *Hoxb5* (*b*,*e*) and *Hoxb6* (*c*,*f*) gene expression in cortex (*a*–*c*) or spinal cord (*d*–*f*) NSPCs that were treated with either DMSO or 1, 10, 100 or 1000 nM RA for 48 h. Transcript levels of *Hoxb4–6* genes in DMSO-treated spinal cord NSPCs are approximately 20–50% of those detected in E13.5 spinal cord tissue, but they are more than 1000 times lower in DMSO-treated cortex NSPCs. Furthermore, RA treatments cause significant, dose-dependent upregulation of *Hoxb4–6* genes in spinal cord, but not in cortex NSPCs. Results are shown as the mean of the log_10_-transformed ratio between DMSO-treated or RA-treated NSPCs and E13.5 spinal cord tissue in four to five biological replicates. Error bars show s.e.m. *p ≤ 0.05; **p < 0.01; ***p < 0.001; n.s., non-significant (*p* > 0.05) according to a two-tailed Student's *t*-test performed between DMSO and RA conditions.

In agreement with previous reports [42,43] and with their expression in the developing mouse spinal cord [20,26], transcription of *Hoxb4–9* genes was maintained during *in vitro* culture of spinal cord NSPCs, while it was barely detectable in cortex NSPCs (figure 2*a*–*f*; electronic supplementary material, figure S1*b* and S1*e*). We then investigated whether exogenous RA could alter these transcriptional profiles. In spinal cord NSPCs cultured in control conditions, mRNA levels of *Hoxb4*, *Hoxb5*, *Hoxb6* and *Hoxb9* were approximately 10–50% of those detectable in E13.5 spinal cord tissue (figure 2*d*–*f*; electronic supplementary material, figure S1*e*). Treatments with increasing doses of RA (from 1 nM to 1 µM) for 48 h led to significant upregulation of these genes by all RA doses, with 1 µM RA eliciting expression levels comparable with those detectable in embryonic spinal cord tissue (figure 2*d*–*f*; electronic supplementary material, figure S1*e*). By contrast, transcript levels of *Hoxb4–9* genes were near the limit of real-time RT-PCR detection in mock-treated cortex NSPCs (approx. 10^−3^–10^−4^ relative to their expression in spinal cord tissue) and they were not significantly changed following exposure to exogenous RA (figure 2*a*–*c*; electronic supplementary material, figure S1*b*). To confirm that cortex NSPCs retained a rostral identity after RA treatments, we analysed expression of the anterior neural tube marker *Emx2*, which is specifically expressed in the developing mouse cerebral cortex [3]. As previously reported [42], *in vitro* cultures of cortex NSPCs showed comparable *Emx2* expression levels to those detected in E13.5 telencephalic tissue (electronic supplementary material, figure S1*c*). Consistently with the inability of RA to upregulate *Hoxb* gene transcription in cortex NSPCs, we found no significant effects of RA treatments on *Emx2* expression in these cultures (electronic supplementary material, figure S1*c*).

During postnatal development, NSPCs of the cerebral cortex mostly undergo terminal differentiation, but a limited population of embryonic cortical progenitors contribute to NSPCs persisting within the SVZ neurogenic niche of the adult and the ageing telencephalon [51]. To address whether rostral NSPCs remain resistant to retinoid-dependent caudalization beyond embryonic development, we analysed the effects of RA treatments on *Hoxb* gene expression in NSPC cultures derived from the adult (3 months old) and the ageing (18 months old) mouse SVZ, as previously described [44]. Similar to cortex NSPCs, treatments of adult and aged SVZ NSPCs with high RA doses (1–2 µM) for 48 h robustly upregulated expression of *Rarb*, *Cyp26a1* and *Dhrs3* (electronic supplementary material, figure S2*a*–*f*), whereas *Hoxb4–6* genes continued to be transcribed at the very low levels detectable in mock-treated cultures without significant changes (electronic supplementary material, figure S3*a*–*f*).

Altogether, these results indicate that RA-dependent signal tranduction is functional and can elicit a transcriptional response in both rostral and caudal NSPCs. By contrast, *Hoxb* genes remain responsive to activation of retinoid signalling in spinal cord NSPCs, whereas in telencephalic NSPCs they are locked in a stably silenced, RA-unresponsive transcriptional state.

### 3.2. Histone H3 methylation and acetylation are differentially enriched at the level of *Hoxb* RAREs in telencephalic and spinal cord NSPCs

We set out to investigate whether the different sensitivity of *Hoxb* genes to retinoid signalling in rostral and caudal NSPCs is epigenetically regulated. To this aim, we focused on *Hoxb4*, *Hoxb5* and *Hoxb6* genes, since their expression in the hindbrain/spinal cord region is controlled by retinoid signalling acting through previously characterized RAREs located upstream of and downstream from *Hoxb4* [19,20,26]. In particular, one RARE (named ENE-RARE) was identified approximately 3 kb downstream from *Hoxb4*, and two more (named B4U-RARE and DE-RARE) roughly 4 and 8 kb upstream of *Hoxb4*, respectively (electronic supplementary material, figure S4; [19,20,26]). We then analysed the levels of key epigenetic modifications around these RAREs.

Previous studies have shown that, in embryonic stem cells (ESCs), *Hox* genes are transcriptionally silent and their chromatin is marked by extensive trimethylation of lysine 27 in histone H3 (H3K27me3), together with limited trimethylation of lysine 4 (H3K4me3) and acetylation of lysines 9 and 14 (H3K9/14ac) [23–25]. Upon ESC differentiation in the presence of exogenous RA, *Hox* genes are upregulated, along with progressive erasure of H3K27me3 and increased deposition of H3K4me3 and H3K9/14ac [23–25]. These observations prompted us to verify whether these epigenetic marks may also be involved in the long-term maintenance of *Hox*-off and *Hox*-on transcriptional states in rostral and caudal NSPCs, respectively, and in the selective ability of caudal NSPCs to upregulate *Hoxb* gene expression in response to RA. To address this question, we carried out ChIP assays with cortex and spinal cord NSPC cultures, using anti-H3K27me3, anti-H3K4me3 and anti-H3K9/14ac antibodies, as described in the Material and methods section. Immunoprecipitated DNA was then assayed by real-time PCR using primer pairs surrounding ENE-RARE, DE-RARE and B4U-RARE. This analysis was extended to the genomic regions upstream of *Hoxb4*, *Hoxb5* and *Hoxb6*, by also testing amplicons located approximately 1–2 kb 5' of these genes.

**Figure 3.**
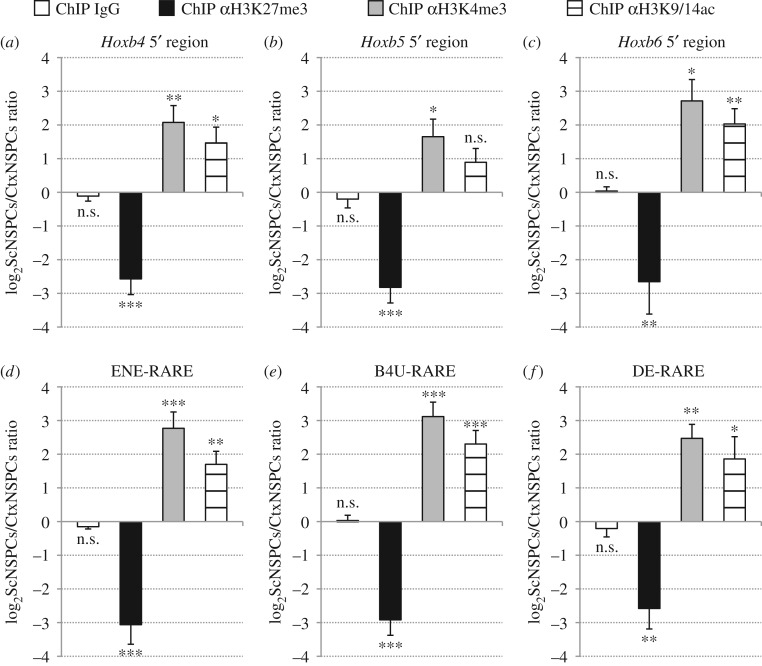
Cortex and spinal cord NSPCs feature differential deposition of H3K27me3, H3K4me3 and H3K9/14ac at the level of both *Hoxb4–6* 5' regions and RAREs. (*a*–*f*) Real-time PCR quantification of ChIP assays in cortex and spinal cord NSPCs using control (IgG), anti-H3K27me3 (αH3K27me3), anti-H3K4me3 (αH3K4me3) or anti-H3K9/14ac (αH3K9/14ac) antibodies. Primer pairs used for real-time PCR target either the genomic regions 5' of *Hoxb4* (*a*), *Hoxb5* (*b*), *Hoxb6* (*c*), or ENE-RARE (*d*), B4U-RARE (*e*), DE-RARE (*f*), as indicated. A significant increase in H3K4me3 and H3K9/14ac levels and significantly lower H3K27me3 levels are detectable in spinal cord NSPCs relative to cortex NSPCs. No changes between spinal cord and cortex NSPCs are detectable following control ChIP (ChIP IgG). Results are shown as the mean of the log_2_-transformed ratio between spinal cord and cortex NSPCs in three to six biological replicates, following normalization to a reference amplicon as described in the Material and methods section. Error bars show s.e.m. *p ≤ 0.05; **p < 0.01; ***p < 0.001; n.s., non-significant (*p* > 0.05) according to a two-tailed Student's *t*-test performed between spinal cord and cortex NSPC samples.

With these assays, we found a significant enrichment of the repressive H3K27me3 mark in cortex NSPCs in comparison with spinal cord NSPCs at the level of both *Hoxb4–6* 5' regions (figure 3*a*–*c*) and ENE-RARE, B4U-RARE and DE-RARE (figure 3*d*–*f*). By contrast, the activating H3K4me3 and H3K9/14ac marks were significantly enriched in the same genomic sequences of spinal cord NSPCs (figure 3*a*–*f*). No significant differences between cortex and spinal cord NSPCs were detected when samples were immunoprecipitated with a control IgG (figure 3*a*–*f*). Since *Hoxb* genes remain transcriptionally inactive and unresponsive to retinoid signalling in adult and aged SVZ NSPCs, we examined whether they retained an epigenetic signature similar to that of embryonic cortical NSPCs. We carried out ChIP assays with anti-K27me3 and anti-K4me3 antibodies on adult and aged SVZ NSPC cultures and found that the overall enrichment of these modifications was comparable with that detected in cortex NSPCs, both at the level of the *Hoxb4* 5' region (electronic supplementary material, figure S5*a*–*c*) and ENE-RARE (electronic supplementary material, figure S5*d*–*f*).

These results suggest that the levels of H3K27me3, H3K4me3 and H3K9/14ac deposition in *Hoxb* chromatin, including key RAREs, may underlie the ability of NSPCs to maintain gene expression profiles and transcriptional responses to retinoid signalling matching their rostrocaudal location.

### 3.3. Retinoid signalling can enhance H3K4me3 and H3K9/14ac deposition in *Hoxb* chromatin of spinal cord NSPCs, but cannot alter its epigenetic signature in telencephalic NSPCs

Previous studies have shown that RA treatments of ESCs cause profound epigenetic changes in the *Hoxb* cluster, by promoting deposition of H3K4me3 and H3K9/14ac at the expense of H3K27me3 [23–25]. We then investigated whether exposure of rostral and caudal NSPCs to exogenous RA could alter the levels of these epigenetic marks in *Hoxb4–6* chromatin.

**Figure 4.**
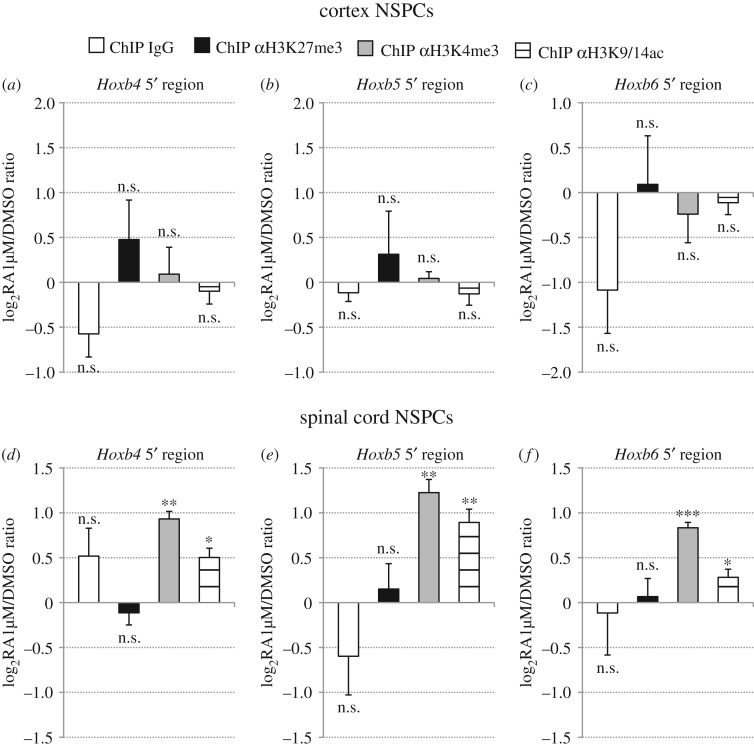
Retinoid signalling promotes H3K4me3 and H3K9/14ac deposition in *Hoxb4–6* 5' regions of spinal cord NSPCs, but does not alter H3K4me3, H3K27me3 and H3K9/14ac levels in *Hoxb4–6* 5' regions of cortex NSPCs. (*a*–*f*) Real-time PCR quantification of ChIP assays in cortex (*a*–*c*) and spinal cord (*d*–*f*) NSPCs that were treated with either DMSO or 1 µM RA for 48 h, using control (IgG), anti-H3K27me3 (αH3K27me3), anti-H3K4me3 (αH3K4me3) or anti-H3K9/14ac (αH3K9/14ac) antibodies. Primer pairs used for real-time PCR target the genomic regions 5' of *Hoxb4* (*a*,*d*), *Hoxb5* (*b*,*e*) or *Hoxb6* (*c*,*f*). A significant increase in H3K4me3 and H3K9/K14ac levels is detectable in RA-treated spinal cord NSPCs relative to DMSO-treated cultures, while H3K27me3 levels are not affected. H3K4me3, H3K27me3 and H3K9/K14ac levels do not show significant differences following RA treatments of cortex NSPCs. No changes between RA-treated and DMSO-treated samples are detectable following control ChIP (ChIP IgG). Results are shown as the mean of the log_2_-transformed ratio between RA-treated and DMSO-treated NSPCs in three to four biological replicates, following normalization to the reference amplicon. Error bars show s.e.m. *p ≤ 0.05; **p < 0.01; ***p < 0.001; n.s., non-significant (*p* > 0.05) according to a two-tailed Student's *t*-test performed between RA-treated and DMSO-treated samples.

**Figure 5.**
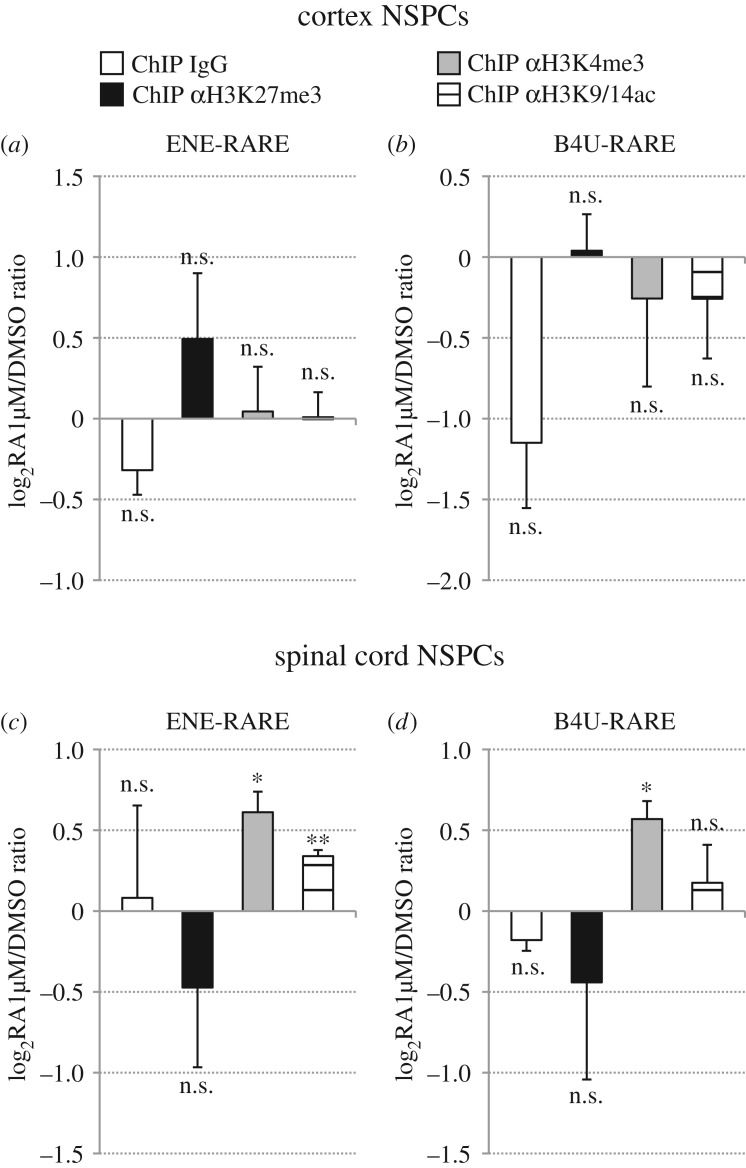
Retinoid signalling promotes H3K4me3 and H3K9/14ac deposition in *Hoxb* RAREs of spinal cord NSPCs, but does not alter H3K4me3, H3K27me3 and H3K9/14ac levels in *Hoxb* RAREs of cortex NSPCs. (*a*–*f*) Real-time PCR quantification of ChIP assays in cortex (*a*,*b*) and spinal cord (*c*,*d*) NSPCs that were treated with either DMSO or 1 µM RA for 48 h, using control (IgG), anti-H3K27me3 (αH3K27me3), anti-H3K4me3 (αH3K4me3) or anti-H3K9/14ac (αH3K9/14ac) antibodies. Primer pairs used for real-time PCR target ENE-RARE (*a*,*c*) or B4U-RARE (*b*,*d*). A significant increase in H3K4me3 and H3K9/K14ac levels is detectable in RA-treated spinal cord NSPCs relative to DMSO-treated cultures, while H3K27me3 levels are not significantly affected. H3K4me3, H3K27me3 and H3K9/K14ac levels do not show significant differences following RA treatments of cortex NSPCs. No significant changes between RA-treated and DMSO-treated samples are detectable following control ChIP (ChIP IgG). Results are shown as the mean of the log_2_-transformed ratio between RA-treated and DMSO-treated NSPCs in three to four biological replicates, following normalization to the reference amplicon. Error bars show s.e.m. *p ≤ 0.05; **p < 0.01; n.s., non-significant (*p* > 0.05) according to a two-tailed Student's *t*-test performed between RA-treated and DMSO-treated samples.

**Figure 6.**
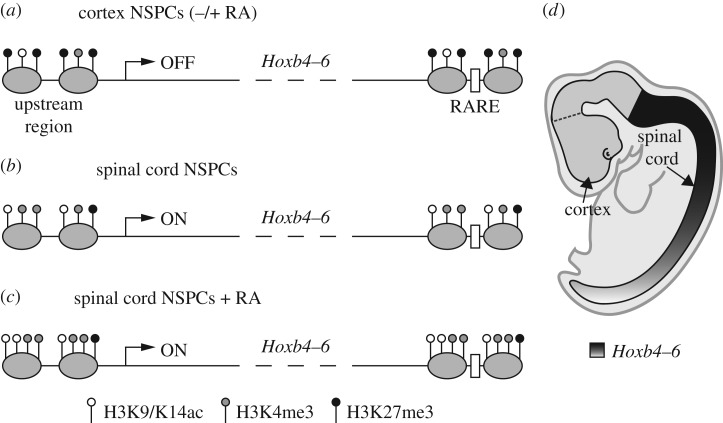
Proposed model of the epigenetic regulation of *Hoxb4–6* expression along the rostrocaudal axis of the neural tube. Rostral (cortex) NSPCs (*a*) undergo increased deposition of H3K27me3 in comparison with caudal (spinal cord) NSPCs (*b*), at the level of *Hoxb4–6* chromatin (here shown for upstream regions and RAREs flanking these genes). By contrast, H3K4me3 and H3K9/14ac are enriched in spinal cord NSPCs in comparison with cortex NSPCs. This differential deposition of activating and repressive epigenetic marks maintains stable repression or activation of *Hoxb4–6* transcription in rostral and caudal NSPCs, respectively. Retinoid signalling cannot alter the epigenetic signature or the repressed transcriptional state of *Hoxb4–6* chromatin in rostral NSPCs (*a*), whereas it can reinforce deposition of activating marks and transcription levels of these genes in caudal NSPCs (*c*). In (*a*–*c*), grey ovals represent nucleosomes, bent arrows represent transcription start sites and white boxes represent RAREs. Panel (*d*) shows a schematic representation of the *Hoxb4–6* expression pattern in the hindbrain/spinal cord region of the developing mouse neural tube. See text for further details.

In these assays, cortex and spinal cord NSPC cultures were treated with 1 µM RA for 48 h, followed by ChIP with anti-H3K27me3, anti-H3K4me3 or anti-H3K9/14ac antibodies and real-time PCR analysis. In agreement with RA-dependent upregulation of *Hoxb* genes in spinal cord NSPCs (figure 2*d*–*f*; electronic supplementary material, figure S1*e*), RA treatments caused in these cells a moderate, but significant increase of H3K4me3 and H3K9/14ac in comparison with mock-treated cultures, at the level of both *Hoxb4–6* 5' regions (figure 4*d*–*f*) and RAREs (figure 5*c*,*d*). No significant changes between DMSO-treated and RA-treated spinal cord NSPC cultures were detectable following ChIP with anti-H3K27me3 antibody or control IgG (figures 4*d*–*f* and 5*c*,*d*). For none of the analysed epigenetic marks, however, did cortex NSPCs show any significant differences between RA-treated cultures and mock-treated cultures (figures 5*a*–*c* and figure 6*a*,*b*).

These results suggest that, at the level of *Hoxb* chromatin, RA can enhance the active epigenetic configuration of caudal NSPCs, whereas rostral NSPCs are insensitive to retinoid signalling both in terms of transcriptional output and deposition of epigenetic marks.

## 4. Discussion

Research on both pluripotent stem cells and tissue-specific stem cells has made significant headway over the last two decades, raising increasing expectations over their use in regenerative medicine in the near future. As far as the CNS is concerned, it is hoped that NSPCs derived from the embryonic or the adult CNS and/or by directed differentiation of pluripotent stem cells *in vitro* may be employed to repair irreversible damage to the neural tissue caused by injury or disease [52,53]. In either case, a key question that needs to be addressed is whether neural progenitors undergoing regional specification due to endogenous patterning cues *in vivo* or to exogenous inductive signals *in vitro* are able to stably retain their positional identities when faced with a different extracellular environment. Since it is well established that neural patterning critically depends on extracellular morphogens [1,54], it is crucial to understand how modifications of morphogen signalling impinge on NSPC positional identities and to shed light on the intrinsic mechanisms acting in NSPCs to interpret extrinsic signals at different developmental stages and CNS locations.

Previous studies have suggested that NSPCs derived from different areas of the mouse fetal CNS (E13.5–14.5) along the rostrocaudal axis maintain their positional identities *in vitro*, as shown by expression profiles of key transcription factors in NSPC cultures that remain consistent with gene expression patterns *in vivo* [42,43]. In this study, we have challenged the intrinsic ability of region-specific NSPCs to retain their positional identities by exposing them to exogenous RA, a morphogen playing a major role in neuroectoderm rostrocaudal patterning [9,10]. In vertebrate embryos exposed to RA during gastrulation, the anterior expression boundaries of *Hox* genes and other posterior neural markers are shifted anteriorly at the expense of rostrally expressed genes, and anterior development is suppressed [17,27]. *In vitro* models of early embryonic progenitors, such as ESCs or embryonic carcinoma cells, respond to exogenous RA applied in the context of neuroectoderm differentiation protocols by efficiently activating *Hox* gene expression [22–25]. Therefore, pluripotent progenitors and/or early neural progenitors arising from them are highly competent in retinoid-driven posteriorization.

By contrast, we demonstrate that NSPCs derived from the developing neural tube at E13.5, when rostrocaudal patterning has already taken place, display distinct, region-specific responses to retinoid signalling. In particular, NSPCs derived from the anterior neural tube were extremely resistant to the caudalizing activity of retinoids and retained rostral gene expression profiles even when exposed to high doses of exogenous RA. Mean levels of retinoid pathway activation were robustly increased by exogenous RA both in rostral and in caudal NSPCs, indicating that retinoid signalling is functional in both cell types, although we cannot exclude differences in the proportion of RA-responsive cells in rostral and caudal NSPC cultures. Thus, anterior positional identities are very labile during early neural patterning [17,27], but subsequently become intrinsically stable and long-lasting, as shown by continued lack of *Hoxb* gene response to high RA levels in rostral NSPCs derived from adult or aged mice. These results strengthen previous observations of stage-dependent reduction in the sensitivity of anterior development to exogenous retinoids in vertebrate embryos [17,28]. Notably, analysis of NSPCs derived from posterior regions of the developing neural tube showed that caudal NSPCs retained not only autonomous expression of *Hoxb4–9* genes *in vitro* as previously described [42,43], but also prolonged competence to upregulate their transcription levels in response to RA. The effects of RA were dose-dependent, eliciting peak levels of *Hoxb* expression at the highest doses used for these assays (1 µM). Significant *Hoxb4–9* upregulation, however, was already detectable at the lowest RA concentration tested (1 nM), which is below the estimated physiological range *in vivo* (2–100 nM [55,56]). This suggests that the continued ability of exogenous RA to promote *Hoxb* expression in caudal neural progenitors is not due to artificial effects of non-physiological RA levels, but reflects genuine responsiveness of *Hoxb* loci to retinoid signalling in NSPCs of the posterior CNS well beyond early rostrocaudal patterning. Previous reports have shown that, in developing mouse embryos, retinoid signalling is required until E11.5 to establish the correct anterior boundaries of *Hoxb* genes in the neural tube [23,26]. Our results indicate that, in caudal NSPCs, *Hoxb4–9* genes remain responsive to RA up to at least E13.5. Though not absolutely necessary for *Hoxb* gene expression, this prolonged window of responsiveness to retinoids and possibly other caudalizing signals may be required to maintain proper *Hox* transcriptional levels at different rostrocaudal levels.

Over the last two decades, a large number of studies have focussed on *Hox* genes as an experimental paradigm to investigate the epigenetic regulation of gene expression during embryogenesis. Extensive efforts have been made in understanding how chromatin modifications of *Hox* clusters can contribute to stage- and position-dependent regulation of *Hox* gene expression and embryonic patterning. Breakthrough work employing mouse and human ESC cultures has suggested that, in these pluripotent cells, *Hox* gene chromatin features opposite epigenetic marks. Whereas deposition of the repressive mark H3K27me3 was found throughout *Hox* clusters, focal deposition of the activating marks H3K4me3 and H3K9/14ac was observed at the level of *Hox* gene transcription start sites [23,24,57]. Whether this bivalent epigenetic configuration is also a defining feature of *Hox* loci in pluripotent cells *in vivo* remains currently unclear [58]. Remarkably, however, RA treatments during ESC differentiation towards the neural lineage were able to dramatically reshape the distribution of these epigenetic marks, causing H3K27me3 erasure across *Hox* clusters and increased levels and spread of H3K4me3 and H3K9/14ac deposition at *Hox* regulatory regions [23–25]. Supporting the importance of these epigenetic modifications in *Hox* gene regulation, landmark studies showed that, in forebrain explants dissected from E10.5 mouse embryos, *Hox* clusters displayed increased levels of H3K27me3 deposition in comparison to ESCs and were devoid of H3K4me3 marks [57,59]. By contrast, in tissue samples dissected from caudal embryonic regions between E8.5 and E10.5, sequential activation of *Hox* genes from the 3' end to the 5' end of the cluster was accompanied by progressive erasure of H3K27me3 and deposition of H3K4me3 and H3K9/14ac along the same direction [57,59,60]. Altogether, these observations provide compelling evidence that the switch from H3K27me3-enriched chromatin to H3K4me3 and H3K9/14ac enrichment is a crucial step in the transcriptional activation of *Hox* genes. They also suggest that these opposite epigenetic signatures may be subsequently employed to maintain stable repression or activation of *Hox* clusters in anterior and posterior embryonic regions, with the bivalent configuration found in ESCs possibly representing an intermediate, initial state in which *Hox* genes are silent but poised for activation.

Our analysis of the epigenetic profiles of *Hoxb* genes in rostral and caudal NSPCs supports the idea that H3K27me3, H3K4me3 and H3K9/14ac may continue to play an important role during the maintenance phase of *Hox* gene neural expression patterns. As summarized in figure 6, we found that genomic sequences flanking *Hoxb4–6* genes, including RAREs controlling their expression in the neural tube, were clearly enriched for H3K27me3 marks in rostral NSPC cultures in comparison with caudal NSPCs, whereas H3K4me3 and H3K9/14ac marks showed opposite profiles. While lacking the power of high-throughput approaches, our results usefully complement previous data obtained with dissected explants from head and trunk regions of developing mice [57,59,61,62]. By taking advantage of culture conditions allowing symmetric NSPC self-renewal *in vitro* [40], our work provides comparison of nearly homogeneous populations of NSPCs, rather than tissue fragments where neural progenitors coexist with differentiated cells and non-neural cell types. Moreover, our analysis covers later developmental stages than previously described and extends up to adulthood and ageing. We show that region-specific epigenetic profiles of *Hoxb* genes in NSPCs are very stable, since they were maintained both during embryonic development and adult life *in vivo* and after long-term passaging *in vitro*. Finally, our work investigates for the first time the effects of retinoid signalling on the epigenetic signature of *Hox* genes in region-specific NSPCs, a task previously carried out only in differentiating pluripotent cells. In contrast with the dramatic remodelling of *Hox* chromatin elicited in ESCs [23–25], RA treatments were utterly unable to modify the levels of H3K27me3, H3K4me3 and H3K9/14ac in rostral NSPCs from E13.5 embryos. Various mechanisms have been proposed to explain temporal and/or positional differences in the competence of progenitor cells to respond to morphogen signalling during neural development [63,64]. We speculate that resistance of rostral neural progenitors to retinoid-driven posteriorization may be linked to disrupted interactions between retinoid signalling and the epigenetic machinery at the level of *Hox* chromatin. RA treatments, however, could enhance deposition of H3K4me3 and, to a lesser extent, H4K9/14ac in caudal NSPCs, suggesting prolonged cross-talk between retinoid signalling and epigenetic regulators of *Hox* chromatin during posterior neural development.

A crucial area of investigation that remains relatively unexplored is related to the respective roles played by different histone modifications and chromatin-modifying complexes in controlling the responsiveness of *Hox* loci to retinoid signalling during neural development. Some initial progress in this direction has been made by analysing the transcriptional response of *Hox* genes to exogenous RA in ESCs or mouse embryos lacking specific epigenetic activities. For example, abrogation of either the H3K27-demethylase UTX or the H3K9-acetyltransferase MOZ hampered RA-driven activation of *Hox* gene expression in differentiating ESCs [55,56,65]. Conversely, loss of components of Polycomb repressive complex 1 (PRC1), a transcriptional repressor complex that may be recruited both via H3K27me3 deposition and independently of it [66], caused premature *Hox* gene response to RA treatments in differentiating ESC cultures and in developing embryos [65,67]. Clearly, much more work is needed in order to understand how the interactions between extracellular signalling pathways and the intracellular epigenetic machinery are modified during development to allow stage- and position-dependent regulation of patterning genes. In recent years, pluripotent stem cell culture systems have allowed steady progress in elucidating the molecular events underlying the early steps of neural patterning [54]. We propose that NSPC *in vitro* systems, due to their robust, simple, highly homogeneous culture conditions [40] and, as shown in this study, their stable epigenetic memory of rostrocaudal positional identities, can represent an excellent experimental paradigm for dissecting the molecular mechanisms controlling maintenance of gene expression programmes and region-specific cell fates.

## Supplementary Material

Supplementary material for the article “Transcriptional response of Hoxb genes to retinoid signalling is regionally restricted along the neural tube rostrocaudal axis”

## Data accessibility

Datasets used for figure preparation are available from the Dryad Digital Repository: http://dx.doi.org/10.5061/dyrad.d22bq [68].
