# Supplementary material for: Transcriptional response of Hoxb genes to retinoid signalling is regionally restricted along the neural tube rostrocaudal axis
Source: R Soc Open Sci. 2017 Apr 5;4(4):160913. doi: 10.1098/rsos.160913 (PMC5414248; doi:10.1098/rsos.160913)
Supplement: Supplementary material for the article “Transcriptional response of Hoxb genes to retinoid signalling is regionally restricted along the neural tube rostrocaudal axis” [file rsos160913supp1.pdf]

SUPPLEMENTARY FIGURE LEGENDS

Figure S1. Retinoid signalling is functional in cortex NSPCs but it cannot alter expression of rostrocaudal markers.

(A-E) Real-time RT-PCR quantification of gene expression in cortex **(A-C)** or spinal cord **(D, E)** NSPCs that were treated with either DMSO or 1, 10, 100 or 1000 nM RA for 48 hours. RA treatments efficiently upregulate the RA-responsive gene *Cyp26a1* **(A, D)** in both cell types and they also cause significant, dose-dependent increase of *Hoxb9* expression in spinal cord NSPCs **(E)**. Transcript levels of *Hoxb9* are nearly 1000 times lower in DMSO-treated cortex NSPCs in comparison with spinal cord NSPCs and they are not significantly changed by exogenous RA **(B)**. Expression levels of the rostral neural marker *Emx2* in cortex NSPCs are comparable to those detected in E13.5 telencephalic tissue and they are not significantly affected by RA treatments **(C)**. Results are shown as the mean of the Log_{10}-transformed ratio between DMSO-treated or RA-treated NSPCs and either E13.5 spinal cord tissue **(A, B, D, E)** or E13.5 telencephalic tissue **(C)** in four to five biological replicates. Error bars show s.e.m. *, $p \leq 0.05$; **, $p < 0.01$; ***, $p < 0.001$; ns, non-significant ($p > 0.05$) according to a two-tailed Student's t-test performed between DMSO and RA conditions.

Figure S2. Retinoid signalling is functional in adult and aged SVZ NSPCs and it can be efficiently activated by exogenous RA.

(A-F) Real-time RT-PCR quantification of gene expression in adult **(A-C)** or aged **(D-F)** SVZ NSPCs that were treated with either DMSO or 1-2 μM RA for 48 hours, showing that RA treatments efficiently upregulate the RA-responsive genes *Rarb*, *Cyp26a1* and *Dhrs3* in both cell types. Results are shown as the mean of the Log_{10}-

transformed ratio between DMSO-treated or RA-treated NSPCs and E13.5 spinal cord tissue in three biological replicates. Error bars show s.e.m. *, $p \leq 0.05$; **, $p < 0.01$; ***, $p < 0.001$ according to a two-tailed Student's t-test performed between DMSO and RA conditions.

Figure S3. *Hoxb4-6* genes are not responsive to retinoid signalling in adult or aged SVZ NSPCs.

(A-F) Real time RT-PCR quantification of *Hoxb4*, **(A, D)**, *Hoxb5* **(B, E)** and *Hoxb6* **(C, F)** expression in adult **(A-C)** or aged **(D-F)** SVZ NSPCs that were treated with either DMSO or 1-2 µM RA for 48 hours. Transcript levels of *Hoxb4-6* genes in SVZ NSPCs are more than 100 times lower than in E13.5 spinal cord tissue, and they are not significantly changed following RA treatments. Results are shown as the mean of the Log_{10}-transformed ratio between DMSO-treated or RA-treated NSPCs and E13.5 spinal cord tissue in three biological replicates. Error bars show s.e.m. ns, non-significant ($p > 0.05$) according to a two-tailed Student's t-test performed between DMSO and RA conditions.

Figure S4. Diagram of mouse *Hoxb4-Hoxb6* genomic region.

Schematic representation of a 26700 base pairs genomic region on mouse chromosome 11, including *Hoxb4*, *Hoxb5* and *Hoxb6* genes, which is based on Ensembl version 85 released in July 2016. Exon coding regions are indicated by black-filled boxes, exon untranslated regions by white-filled boxes and introns by black horizontal lines. Vertical lines indicate the locations of DE-RARE, B4U-RARE and ENE-RARE.

Figure S5. Deposition of H3K27me3 and H3K4me3 at the level of both *Hoxb4* 5' region and ENE-RARE is comparable in E13.5 cortex NSPCs, adult SVZ NSPCs and aged SVZ NSPCs.

(A-F) Real-time PCR quantification of ChIP assays in embryonic cortex NSPCs **(A, D)**, adult SVZ NSPCs **(B, E)** and aged SVZ NSPCs **(C, F)** using control (IgG), anti-H3K27me3 (αH3K27me3) or anti-H3K4me3 (αH3K4me3) antibodies. Primer pairs used for real-time PCR target *Hoxb4* 5' region **(A-C)** or ENE-RARE **(D-F)**. SVZ NSPCs feature comparable levels of H3K27me3 and H3K4me3 relative to cortex NSPCs. No differences between SVZ and cortex NSPCs are detectable following control ChIP (ChIP IgG). Results are shown as the mean of the Log_2-transformed ratio between immunoprecipitated samples and input samples in four to five biological replicates, following normalization to a reference amplicon as explained in the Material and methods section. Error bars show s.e.m.

Figure S1

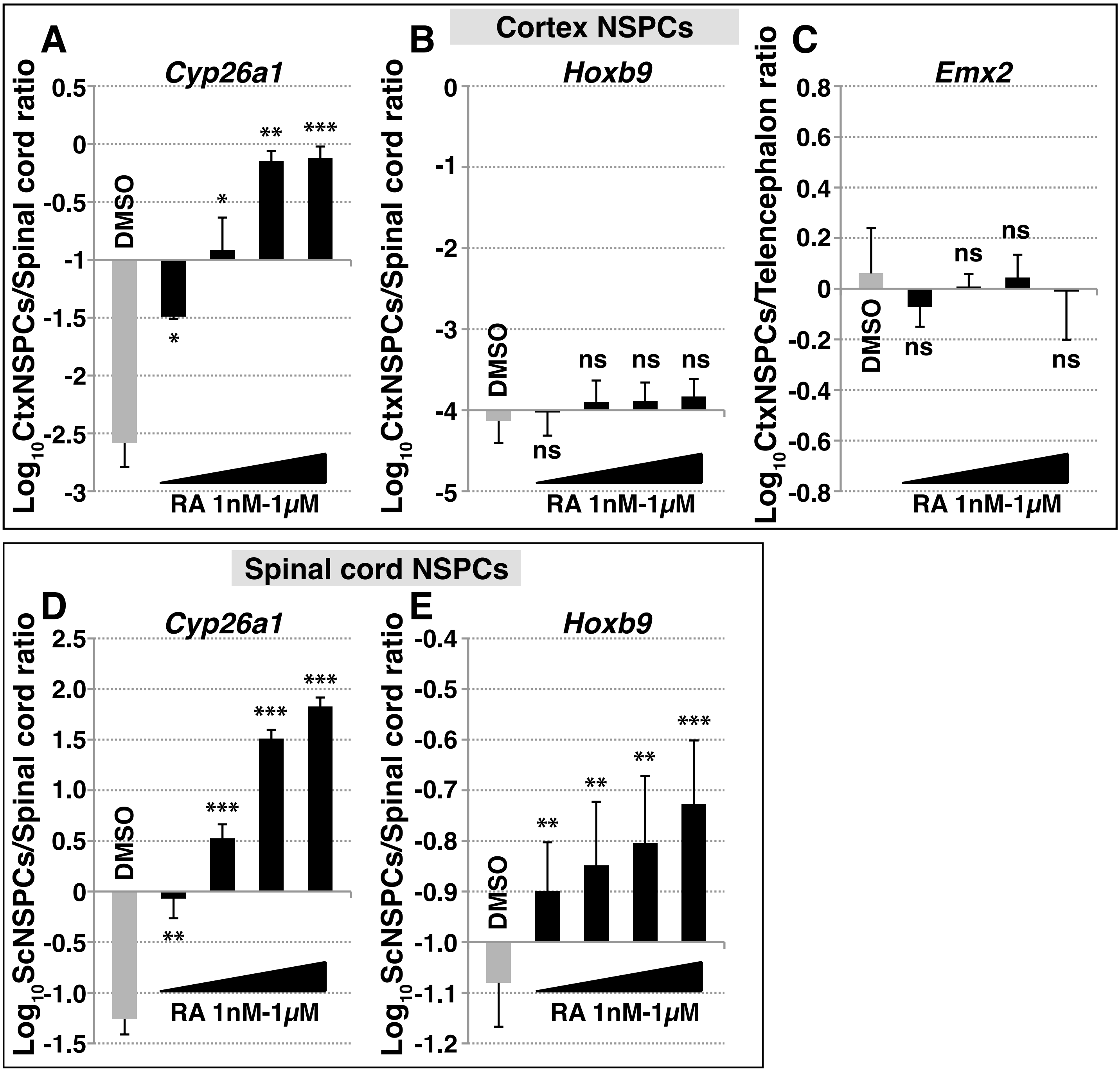
Cortex NSPCs
A
Cyp26a1
Log10CtxNSPCs/Spinal cord ratio
0.5
0
-0.5
-1
-1.5
-2
-2.5
-3
DMSO
*
*
**
***
RA 1nM-1μM
B
Hoxb9
Log10CtxNSPCs/Spinal cord ratio
0
-1
-2
-3
-4
-5
DMSO
ns
ns
ns
ns
RA 1nM-1μM
C
Emx2
Log10CtxNSPCs/Telencephalon ratio
0.8
0.6
0.4
0.2
0
-0.2
-0.4
-0.6
-0.8
DMSO
ns
ns
ns
ns
RA 1nM-1μM
Spinal cord NSPCs
D
Cyp26a1
Log10ScNSPCs/Spinal cord ratio
2.5
2.0
1.5
1.0
0.5
0
-0.5
-1.0
-1.5
DMSO
**
***
***
***
RA 1nM-1μM
E
Hoxb9
Log10ScNSPCs/Spinal cord ratio
-0.4
-0.5
-0.6
-0.7
-0.8
-0.9
-1.0
-1.1
-1.2
DMSO
**
**
**
***
RA 1nM-1μM

Figure S2

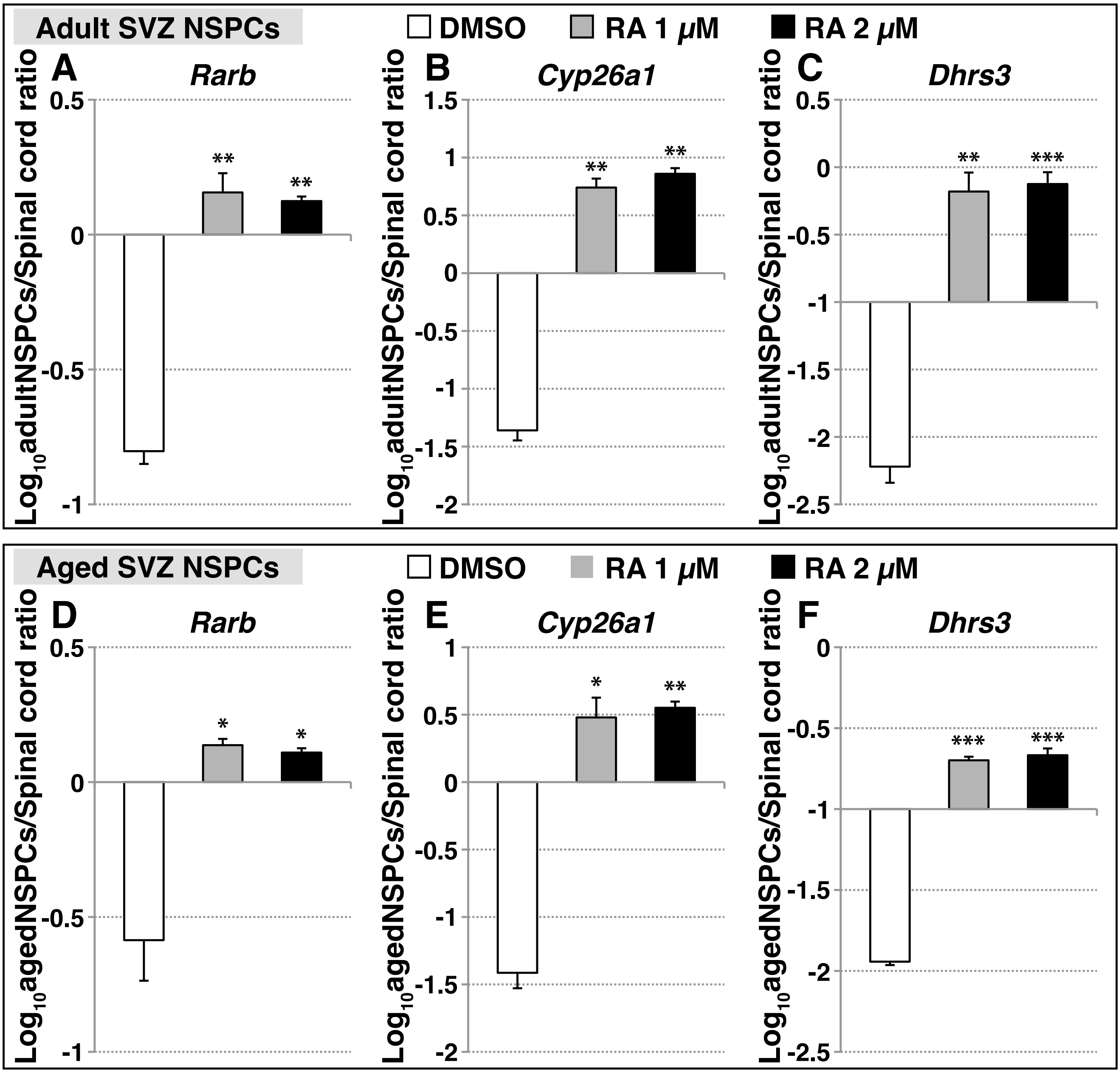

Adult SVZ NSPCs
DMSO
RA 1 μM
RA 2 μM
A
Rarb
B
Cyp26a1
C
Dhrs3
Log10adultNSPCs/Spinal cord ratio
Aged SVZ NSPCs
D
Rarb
E
Cyp26a1
F
Dhrs3
Log10agedNSPCs/Spinal cord ratio

Figure S3

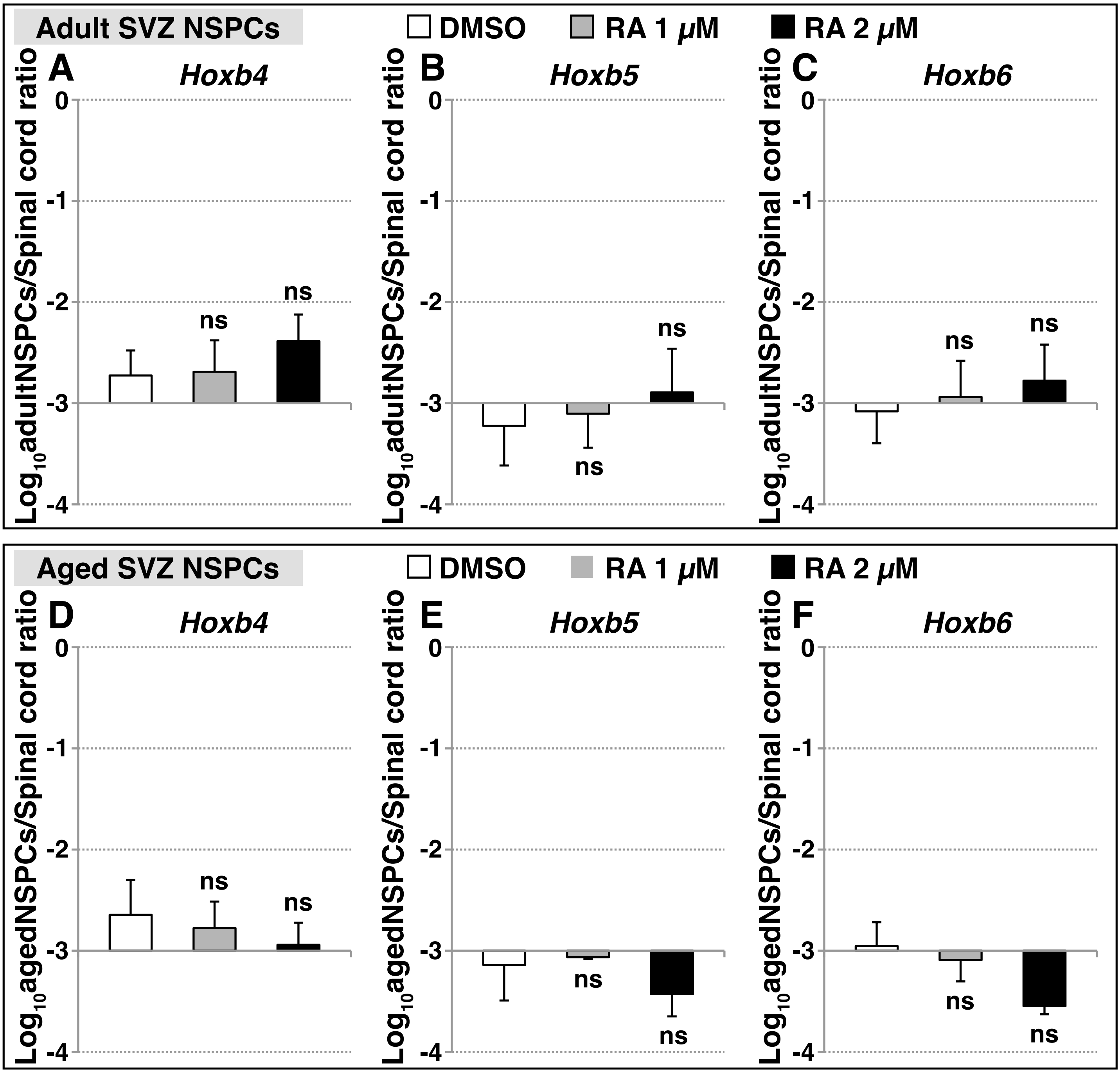

Adult SVZ NSPCs
DMSO
RA 1 μM
RA 2 μM
A
Hoxb4
B
Hoxb5
C
Hoxb6
Log10adultNSPCs/Spinal cord ratio
0
-1
-2
-3
-4
ns
Aged SVZ NSPCs
D
Hoxb4
E
Hoxb5
F
Hoxb6
Log10agedNSPCs/Spinal cord ratio

Created with SnapGene®

Figure S4

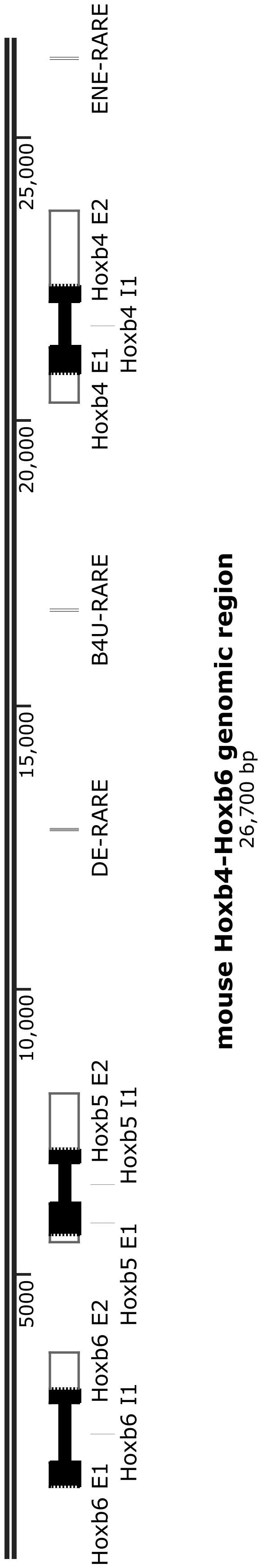

Figure S5

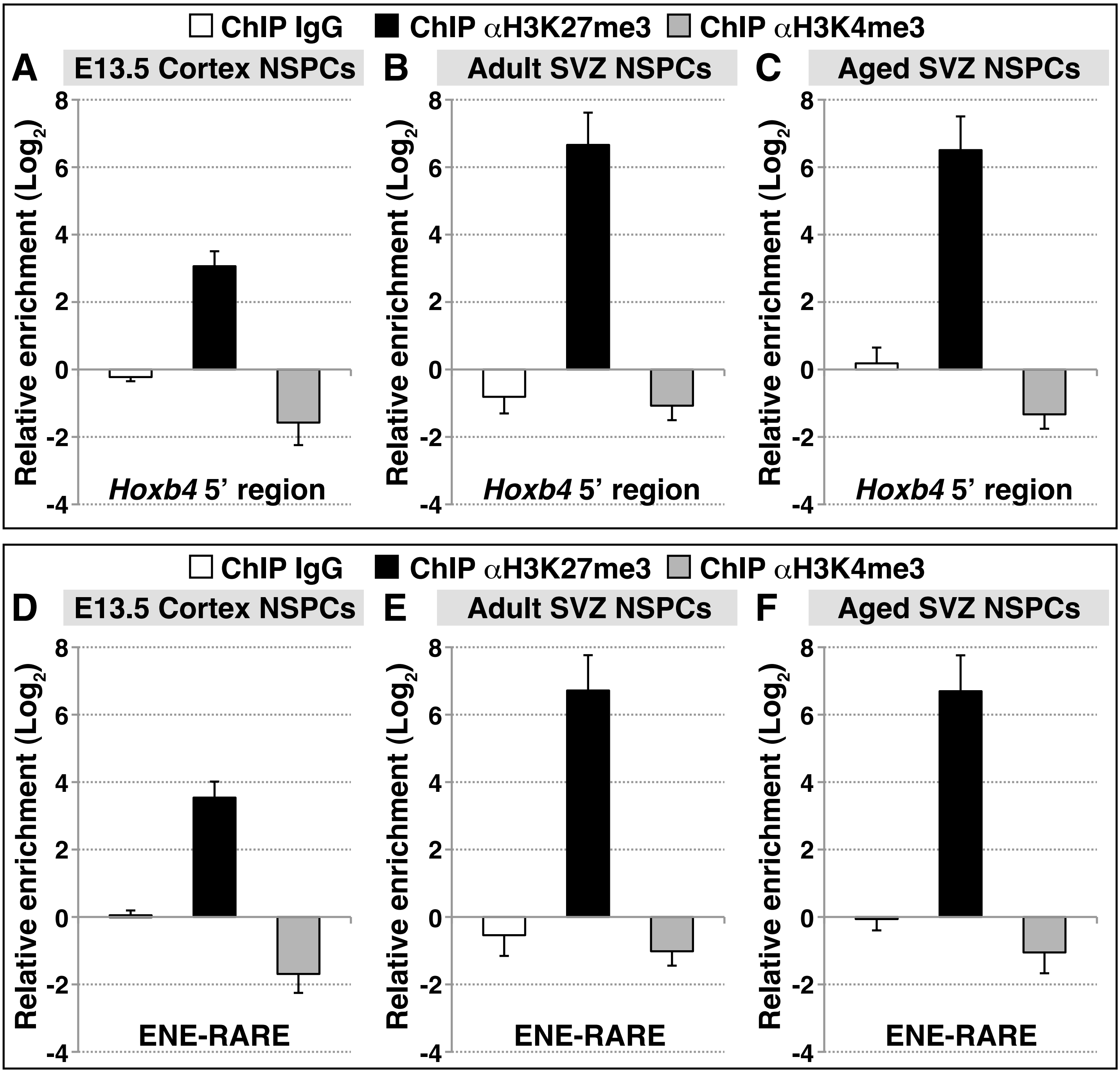

ChIP IgG
ChIP αH3K27me3
ChIP αH3K4me3
A
E13.5 Cortex NSPCs
B
Adult SVZ NSPCs
C
Aged SVZ NSPCs
Relative enrichment (Log2)
8
6
4
2
0
-2
-4
Hoxb4 5' region
D
E13.5 Cortex NSPCs
E
Adult SVZ NSPCs
F
Aged SVZ NSPCs
ENE-RARE

Name	Forward primer (5’-3’)	Reverse primer (5’-3’)
Rarb	TTCCTGGATCAATGCCACCTC	TGACTCCACTGTTCTCCACTG
Dhrs3	CCGTGGACCATGAATATCCT	GCTCCTCAGGTGTGTCTTCC
Hoxb4	AAGTTGCCCAACACCAAGAT	CTTCGGTGCTCGCTGTTC
Hoxb5	GTCGCATGAAGTGGAAGAAAG	GGAAGATTGGAAGGGTCGAG
Hoxb6	CAAACTGCTCAGTGCGTCTC	CTAGGTTCCCCCACAGACCT
Hoxb9	CATGAAGTGGCCAGACTCCT	TACAACCCAGACAGCAGTGG
Eef1a1	AGCTGGCAAAGTCACCAAGT	CCGTTCTTCCACCACTGATT
Rpl19	AGACCAAGGAAGCACGAAAG	GCCGCTATGTACAGACACGA

Table S1. List of primers used in this study for real-time RT-PCR analyses.

Name	Forward primer (5’-3’)	Reverse primer (5’-3’)
Hoxb4 5′ region	AACCCAGAGGACAACATTGC	TGGGTCCAGAGACCTCAGTC
Hoxb5 5′ region	CCAGCGTTGGTTGTGTCTC	ACAACCGAGGCAAGCTTAGA
Hoxb6 5′ region	TTACCTAGGCTGGGGTTTCC	TGCTATTGCTATTTGGCAAGC
DE-RARE	CATCCTGAAAGGGGAAATCA	GGTAATGTATGCGGCAGAGG
B4U-RARE	CAAGTAATGACCTGCGCAAA	TCACGAGGTTCTCGAACATTT
ENE-RAE	CTGAATGTGCTGTCCCCTTT	CCGAGGTCATTTTGTGGTTC
Eef1a1 5′ region	TGCAGCAAAGAAAGGGATGT	CTAATCCACCCCACATGGTC

Table S2. List of primers used in this study for real-time ChIP-PCR analyses.
